# First case report of robot‐assisted radical cystectomy for bladder cancer that developed after salvage radiotherapy following radical prostatectomy for prostate cancer

**DOI:** 10.1002/iju5.12616

**Published:** 2023-07-27

**Authors:** Suguru Oka, Kazushige Sakaguchi, Michikata Hayashida, Shinji Ito, Kazuhiro Kurosawa, Shinji Urakami

**Affiliations:** ^1^ Department of Urology Toranomon Hospital Tokyo Japan; ^2^ Department of Pathology Toranomon Hospital Tokyo Japan

**Keywords:** bladder cancer, prostate cancer, robot‐assisted radical cystectomy

## Abstract

**Introduction:**

Robot‐assisted radical cystectomy for bladder cancer that develops after curative treatment for prostate cancer has not yet been reported.

**Case presentation:**

A 65‐year‐old man underwent radical prostatectomy and received salvage radiotherapy after his postoperative prostate‐specific antigen level failed to decrease. Nine years after radiotherapy, local recurrence and lung/bone metastases were observed, and he was started on androgen deprivation therapy. In the following year, he was diagnosed with nonmuscle invasive bladder cancer. He underwent transurethral resection of the bladder tumor once but had multiple recurrences within 3 months. As hematuria could not be controlled by transurethral surgery, he underwent robot‐assisted radical cystectomy without rectal injury. Since then, there has been no recurrence of either bladder or prostate cancer.

**Conclusion:**

This is the first report of a successful robot‐assisted radical cystectomy for bladder cancer that developed after local salvage radiotherapy following radical prostatectomy for prostate cancer.

**Abbreviations & Acronyms**
ADT: androgen deprivation therapy
NMIBC: nonmuscle invasive bladder cancer
PSA: prostate‐specific antigen
RARC: robot‐assisted radical cystectomy
RT: radiation therapy
SRT: salvage RT
TPE: total pelvic exenteration
TUC: transurethral coagulation
TURBT: transurethral resection of bladder tumor

**Keynote message**

We report the first case of robot‐assisted radical cystectomy that was successfully performed without rectal injury for bladder cancer that developed after salvage radiotherapy following radical prostatectomy for prostate cancer.

## Introduction

Radical cystectomy after treatment for prostate cancer is expected to involve high adhesion due to previous surgery and RT, thereby increasing the difficulty of the procedure and the risk of perioperative complications.^1^ The overall perioperative complication and mortality rates for radical cystectomy in patients without prior RT have been reported to be 49% to 64% and 2% to 3%, respectively,^2^, ^3^, ^4^ whereas patients who underwent radical cystectomy after RT of 60 Gy or more had higher rates of 77% and 14.9%, respectively.^1^ Gontero *et al*. reported that bowel leakage occurred in 6.2% of patients with a history of pelvic radiation undergoing radical cystectomy.^5^ RARC was first reported in 2003.^6^ RARC has been shown to be noninferior to open radical cystectomy in cancer control and complications^7^ and is widely performed in Japan. There are few reports of curative surgical treatment for bladder cancer that developed after SRT for postoperative recurrence of prostate cancer. In the era of open surgery, TPE was performed in such cases.^8^ There are no reports in the laparoscopic and robotic surgery era. We report the first case in which RARC was performed in such a patient without rectal injury.

## Case report

**Fig. 1:**
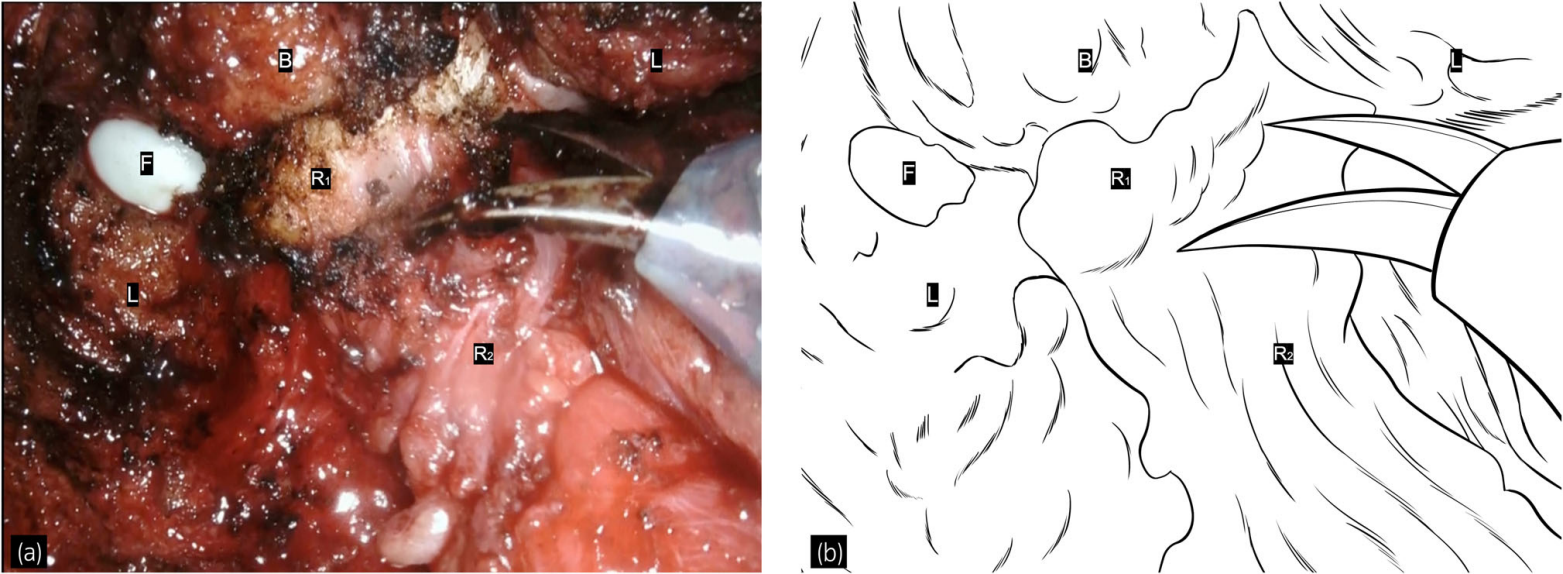
(a) A still image taken during the procedure and (b) the corresponding schematic illustration. It shows a scene in which the bladder and rectal muscle layers are dissected, the levator ani is accessed from the perineal approach side, and a finger is inserted as a guide. The labels are as follows: B, bladder; F, finger; L, levator ani; R1, area where both the bladder and rectal muscle layers are distinctly exposed and laid bare; R2, area where the Denonvilliers' fascia is attached to the anterior rectal surface.

**Fig. 2:**
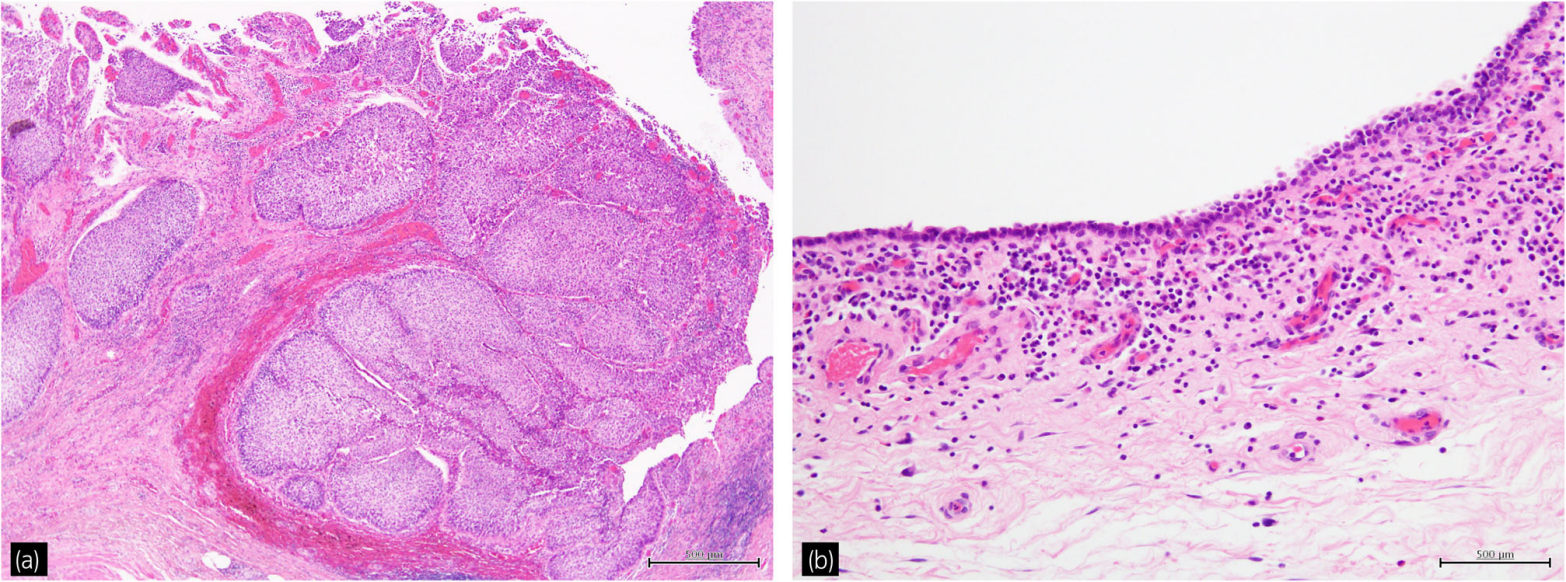
Hematoxylin–eosin staining revealed (a) high‐grade urothelial carcinoma (40×) and (b) general atrophy of the bladder mucosa with a thin urothelial layer, infiltration of lymphocytes and plasma cells in the subepithelial layer, and the presence of fibrosis and angiogenesis (200×).

A 65‐year‐old man with a high PSA of 12.44 ng/mL was diagnosed with prostate cancer. He underwent radical prostatectomy and pathological analysis revealed adenocarcinoma, pT2aN0M0, Gleason score 4 + 4, and positive margins.^9^ Because his postoperative PSA level only decreased to 0.32 ng/mL, local SRT with a total of 70 Gy was administered. After SRT, PSA nadir was 0.02 ng/mL, and serum PSA level was increased. Three years after the RT, bladder hemorrhage due to radiation cystitis was observed, but the bleeding resolved with conservative treatment. In the same year, hyperbaric oxygen therapy was administered. He required hospitalization and treatment for bladder bleeding every year thereafter, and TUC procedures had been required four times in the 10 years after RT. After 9 years of RT, local recurrence, lung and bone metastases were observed. Therefore, ADT was started, and PSA decreased to 0.01 ng/mL. After 11 years of RT, he underwent TUC for bladder hemorrhage again. At that time, a bladder tumor was found, and TURBT was performed the following month. Pathologic results showed urothelial carcinoma, pTa, high grade. Postoperative bleeding was observed, two units of red blood cells were transfused, and TUC was performed 2 weeks after the TURBT. Cystoscopy revealed multiple bladder cancer recurrences 3 months after TURBT. After informed consent was obtained from the patient, the decision was made to perform an immediate RARC, considering the possibility that TURBT would have resulted in uncontrolled bleeding. The operative time was 595 min, and blood loss was 392 cc. The surgeon carefully dissected the space between the bladder and rectum, where the dissected layer was difficult to identify, using digital rectal examination to check its thickness by moving it back and forth from inside the rectum and from side to side. The dissection around the vesicourethral anastomosis was performed while checking the contour of the urethra (Fig. 1). The anterolateral side around the vesicourethral anastomosis was dissected at the position where the levator ani muscle contracts and the ventral side was dissected at the line where the pubic bone is exposed. The DVC was dissected without ligation, and the bladder was removed with little bleeding. The pelvic floor defect was minimal and could be closed as in a typical cystectomy. Since the small intestine and ureter did not appear to be grossly affected by the radiation, the procedure was completed with the usual extracorporeal urinary diversion. Dietary resumption occurred on the second postoperative day. One week postsurgery, a postoperative infection was noted, necessitating a 2‐week course of antibiotics. Aside from this, the patient's recovery proceeded without incident. The highest complication in the Clavien‐Dindo Classification was grade II.^10^ Histopathologic analysis revealed urothelial carcinoma, pTa, high grade (Fig. 2). There was no pathological local recurrence of prostatic adenocarcinoma in resected specimens. The bladder was generally atrophic. There were lymphocyte and plasma cell infiltration, fibrosis, and congestion in the subepithelial layer. One year after surgery, the PSA level of 0.01 ng/mL was maintained with continued ADT. Urine cytology was class II and no recurrence was observed on imaging, indicating no progression of prostate cancer or recurrence of bladder cancer.

## Discussion

We successfully performed a RARC without incurring rectal injury. This is a unique case in which RARC was performed for a patient with NMIBC that was expected to be difficult to cure by TURBT only and was also expected to be difficult to control if vesical bleeding occurred after TURBT and/or intravesical therapy again.

There are several reasons for performing RARC for multiple recurrent NMIBC without prior TURBT. First, the mortality rate of cystectomy for uncontrolled hemorrhagic cystitis is 16%.^11^ Therefore, if there was a high likelihood of uncontrolled bleeding, immediate radical cystectomy would be appealing. Second, this patient did not want TURBT because of pain during bladder irrigation for hemorrhagic cystitis and bleeding after TURBT and chose cystectomy over bladder preservation. A possible serious complication of this surgery is rectal injury. The incidence of anastomotic leakage (25.0% *vs* 13.7%; *p* = 0.019) and the requirement for a permanent stoma (41.0% *vs* 12.4%; *p* < 0.001) were significantly higher in patients who underwent rectal cancer surgery following prior curative treatment for prostate cancer, compared with those who did not receive any prostatic intervention.^12^ There are no reports of radical cystectomy after SRT following radical prostatectomy, but considering the previous reports, it should be recognized that rectal injury is more likely to result in permanent colostomy than routine radical total cystectomy. Therefore, the patient should be informed of the possibility of permanent colostomy before surgery. Significantly, this report acknowledges its limitations as it presents an exceptional case of radical cystectomy, a procedure that deviates from the conventional treatment approach.

## Conclusion

We report the first case of RARC, which was successfully performed without rectal injury for bladder cancer that developed after local SRT following radical prostatectomy for prostate cancer.

## Author contributions

Suguru Oka: Conceptualization; investigation; writing – original draft. Kazushige Sakaguchi: Conceptualization; writing – review and editing. Michikata Hayashida: Writing – review and editing. Shinji Ito: Writing – review and editing. Kazuhiro Kurosawa: Writing – review and editing. Shinji Urakami: Writing – review and editing.

## Conflict of interest

The authors declare no conflict of interest.

## Approval of the research protocol by an Institutional Reviewer Board

Not Applicable.

## Informed consent

Consent to participate and for publication was acquired from the patient.

## Registry and the Registration No. of the study/trial

Not Applicable.
